# The genome sequence of the Sallow moth, *Xanthia icteritia *(Hufnagel, 1766)

**DOI:** 10.12688/wellcomeopenres.21623.1

**Published:** 2024-05-15

**Authors:** Inez Januszczak, David C. Lees, Natural History Museum Genome Acquisition Lab, Darwin Tree of Life Barcoding collective, Wellcome Sanger Institute Tree of Life Management, Samples and Laboratory team, Wellcome Sanger Institute Scientific Operations: Sequencing Operations, Wellcome Sanger Institute Tree of Life Core Informatics team, Tree of Life Core Informatics collective, Darwin Tree of Life Consortium

**Affiliations:** 1 Natural History Museum, London, England, UK

**Keywords:** Xanthia icteritia, Sallow moth, genome sequence, chromosomal, Lepidoptera

## Abstract

We present a genome assembly from an individual male *Xanthia icteritia* (the Sallow moth; Arthropoda; Insecta; Lepidoptera; Noctuidae). The genome sequence is 664.6 megabases in span. Most of the assembly is scaffolded into 31 chromosomal pseudomolecules, including the Z sex chromosome. The mitochondrial genome has also been assembled and is 15.58 kilobases in length. Gene annotation of this assembly on Ensembl identified 18,792 protein coding genes.

## Species taxonomy

Eukaryota; Opisthokonta; Metazoa; Eumetazoa; Bilateria; Protostomia; Ecdysozoa; Panarthropoda; Arthropoda; Mandibulata; Pancrustacea; Hexapoda; Insecta; Dicondylia; Pterygota; Neoptera; Endopterygota; Amphiesmenoptera; Lepidoptera; Glossata; Neolepidoptera; Heteroneura; Ditrysia; Obtectomera; Noctuoidea; Noctuidae; Xyleninae; *Xanthia*; *Xanthia icteritia* (Hufnagel, 1766)(NCBI:txid987434).

## Background

The Sallow, *Xanthia icteritia* (Hufnagel, 1766), sometimes placed in the genus or subgenus *Cirrhia (*Hübner, 1821), of which it is the type species, is a moth of the family Noctuidae. It has a broad distribution, found across the Palaearctic realm mainly in northern Europe, stretching to east Asia, including Japan and Korea ( GBIF Secretariat, 2024). It was recently recorded in the Jammu and Kashmir region, 1600 m) of India ( Riyaz & Sivasankaran, 2022). In Britain it occupies most woodlands, heathlands and marsh-like habitats, usually flying in the autumn, latest in the south ( Randle *et al.*, 2019). The name derives from the initial host plant of the larvae, the catkins of sallow ( *Salix* sp.), with the larvae later feeding on herbaceous plants ( Waring *et al.*, 2017).

With a wingspan of 30 to 40 mm, adults usually have pale yellow forewings, with a yellow fringe, and a dark blotch with a pale centre at the base of the reniform stigma. However, there are many colour morphs, usually strongly marked with purplish brown to forms with plain yellow-orange forewings ( Seitz, 1914). A study by Nozawa and Inari (2005) in Japan showed that the larvae of *Xanthia icteritia* share inflorescence resources with five other insect species, preferring *Salix miyabeana* Seemen over the two other dominant *Salix* studied. They were observed to consume more catkin resources than other potential competitors, feeding on all parts of both male and female inflorescences.

British macro-moths are rapidly declining, with the annual total number of macro-moths caught by Rothamsted Insect Survey (RIS) light trap networks showing a 31% decrease over a 35-year sampling period ( Conrad *et al.*, 2006). The study categorised *Xanthia icteritia* as ‘vulnerable’, with an annual population decrease of 4.8%. This decrease was larger than that of other “ *Xanthia”* species included in the study (Barred Sallow, *X. aurago*, and Pink-Barred Sallow, *X. togata*, both currently placed in the genus *Tiliacea* Tutt, 1896), but lower than the Dusky-Lemon Sallow ( *Xanthia gilvago*) which had an annual decrease of 7%. However, Randle *et al*. (2019) characterised as ‘severe’ the 81% decline in abundance abundance in Great Britain between 1970 and 2016.

We present a chromosomally complete genome sequence for the Sallow moth, *Xanthia icteritia*, based on one specimen collected in Scotland as part of the Darwin Tree of Life Project. This project is a collaborative effort to sequence all named eukaryotic species in the Atlantic Archipelago, encompassing Britain and Ireland. DNA barcode records on BOLD show several species of *Xanthia* Ochsenheimer, 1816, notably *X. ocellaris* (Borkhausen, 1792) and *X. gilvago* (Denis & Schiffermüller, 1775) are genetically closely related to *X. icteritia* and these are frequently placed in the genus *Cirrhia*. This genome could help to clarify phylogenetic relationships in this group of moths and highlights the existing nomenclatural confusion (BOLD accessed 19 July 2023) of *Xanthia* with the genus *Cirrhia* Hübner, 1821.

## Genome sequence report

**Figure 1.**
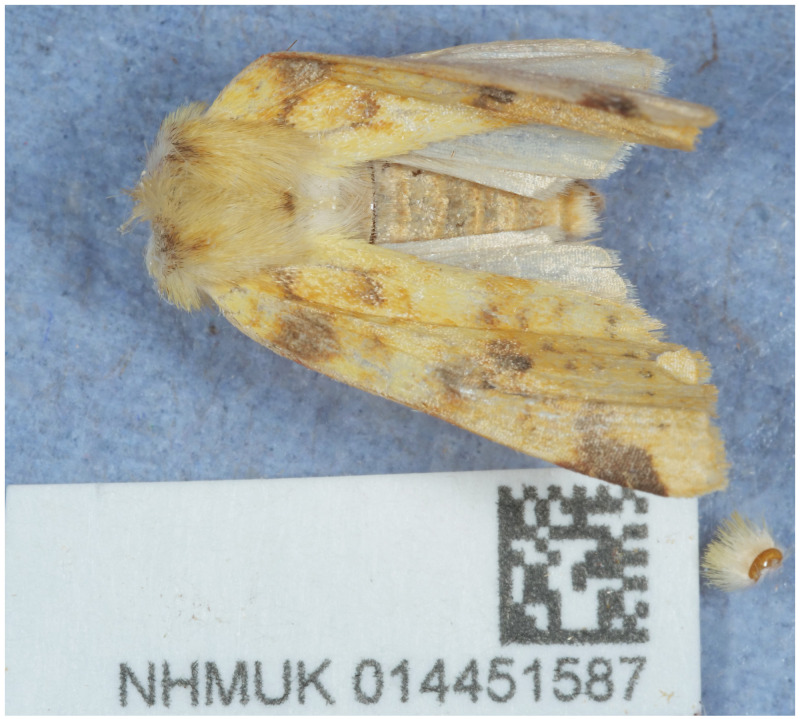
Photograph of the *Xanthia icteritia* (ilXanIcte2) specimen used for genome sequencing.

The genome was sequenced from a male *Xanthia icteritia* ( Figure 1) collected from Beinn Eighe National Nature Reserve, Scotland, UK (57.63, –5.35). A total of 46-fold coverage in Pacific Biosciences single-molecule HiFi long reads was generated. Primary assembly contigs were scaffolded with chromosome conformation Hi-C data. Manual assembly curation corrected 15 missing joins or mis-joins and removed 6 haplotypic duplications, reducing the assembly length by 1.25% and the scaffold number by 19.57%, and increasing the scaffold N50 by 0.23%.

**Figure 2.**
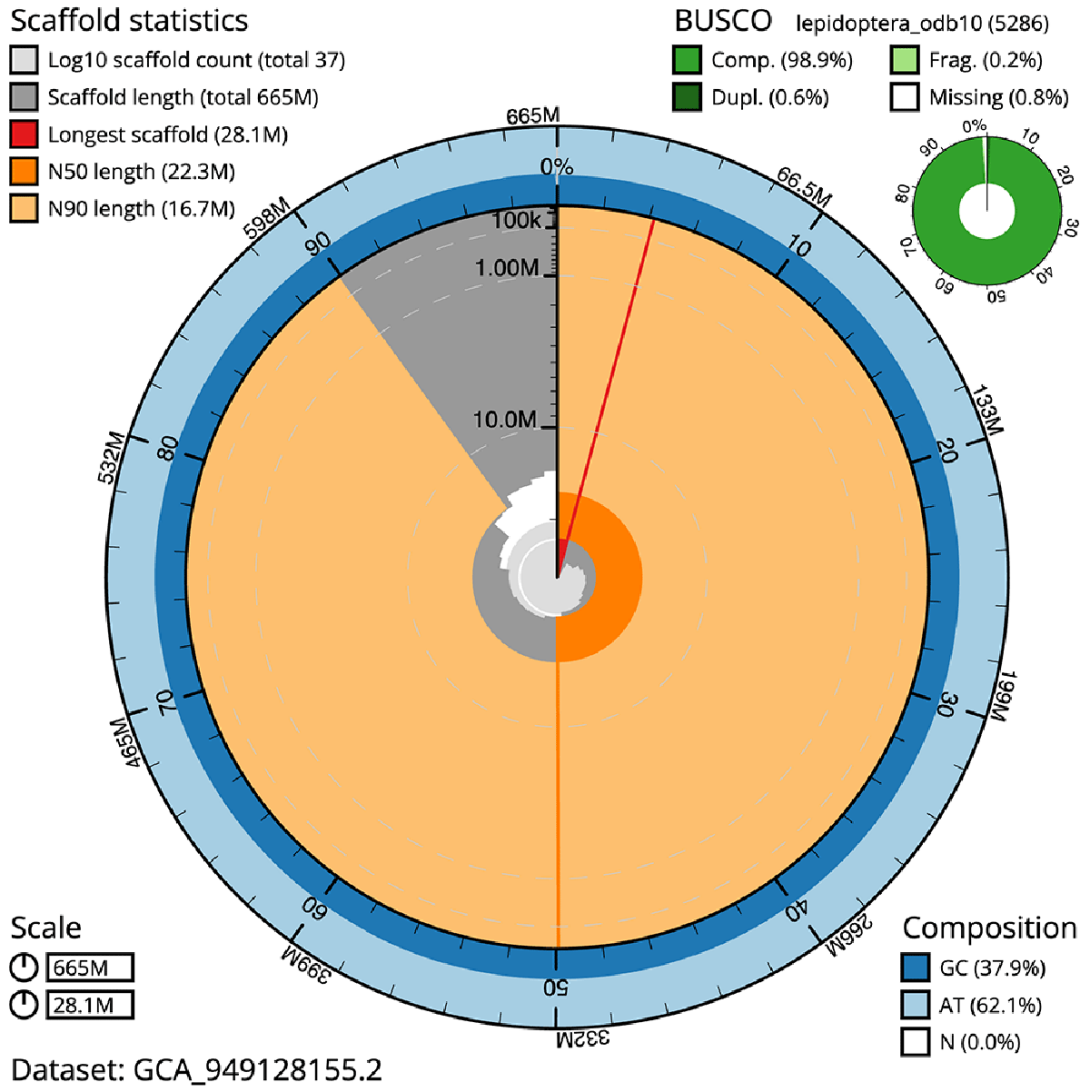
Genome assembly of *Xanthia icteritia*, ilXanIcte2.2: metrics. The BlobToolKit snail plot shows N50 metrics and BUSCO gene completeness. The main plot is divided into 1,000 size-ordered bins around the circumference with each bin representing 0.1% of the 664,638,346 bp assembly. The distribution of scaffold lengths is shown in dark grey with the plot radius scaled to the longest scaffold present in the assembly (28,052,002 bp, shown in red). Orange and pale-orange arcs show the N50 and N90 scaffold lengths (22,252,460 and 16,667,398 bp), respectively. The pale grey spiral shows the cumulative scaffold count on a log scale with white scale lines showing successive orders of magnitude. The blue and pale-blue area around the outside of the plot shows the distribution of GC, AT and N percentages in the same bins as the inner plot. A summary of complete, fragmented, duplicated and missing BUSCO genes in the lepidoptera_odb10 set is shown in the top right. An interactive version of this figure is available at https://blobtoolkit.genomehubs.org/view/Xanthia_icteritia/dataset/GCA_949128155.2/snail.

**Figure 3.**
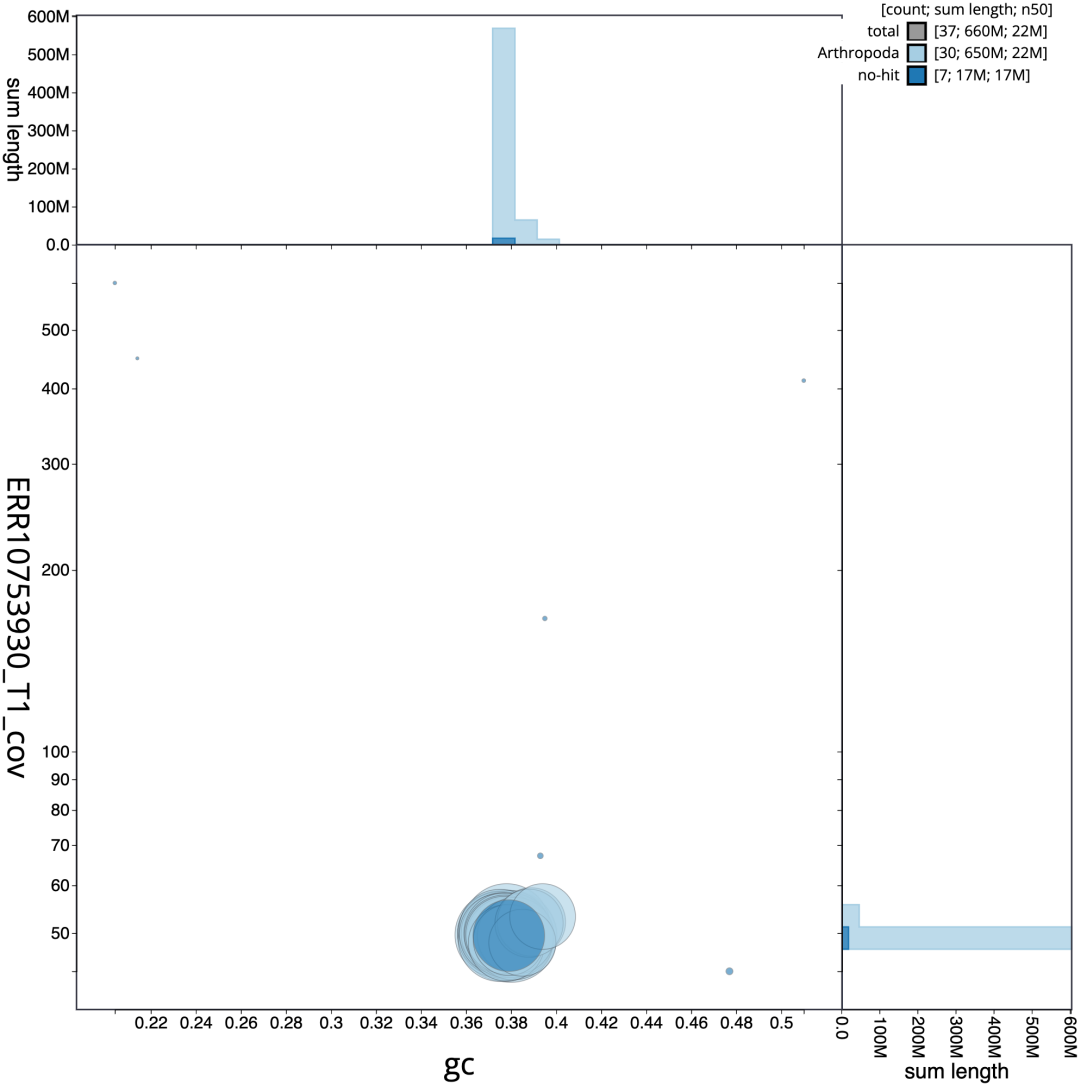
Genome assembly of *Xanthia icteritia*, ilXanIcte2.2: BlobToolKit GC-coverage plot. Sequences are coloured by phylum. Circles are sized in proportion to sequence length. Histograms show the distribution of sequence length sum along each axis. An interactive version of this figure is available at https://blobtoolkit.genomehubs.org/view/Xanthia_icteritia/dataset/GCA_949128155.2/blob.

**Figure 4.**
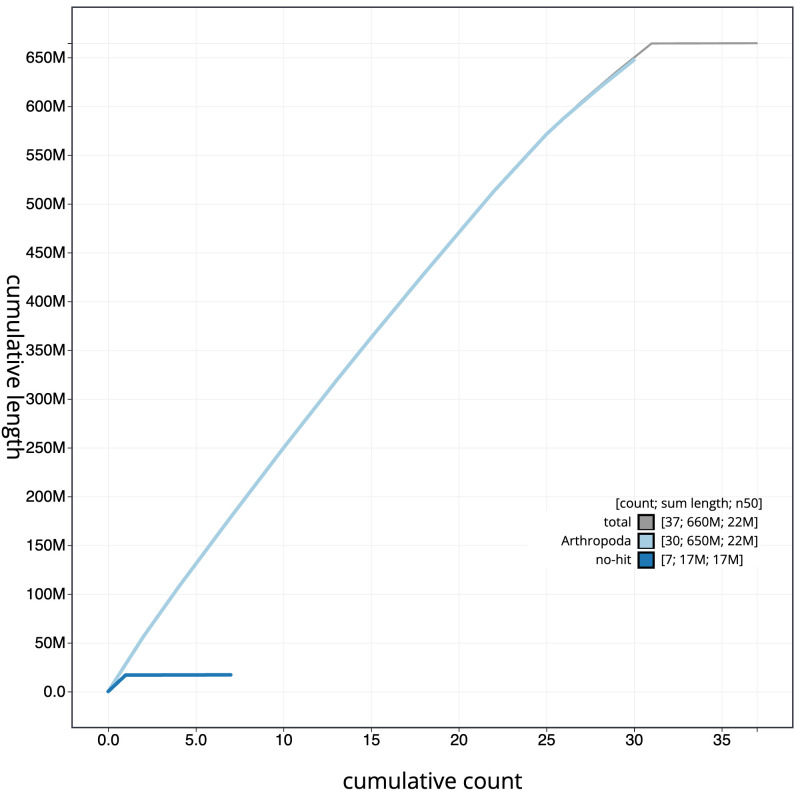
Genome assembly of *Xanthia icteritia*, ilXanIcte2.2: BlobToolKit cumulative sequence plot. The grey line shows cumulative length for all sequences. Coloured lines show cumulative lengths of sequences assigned to each phylum using the buscogenes taxrule. An interactive version of this figure is available at https://blobtoolkit.genomehubs.org/view/Xanthia_icteritia/dataset/GCA_949128155.2/cumulative.

**Figure 5.**
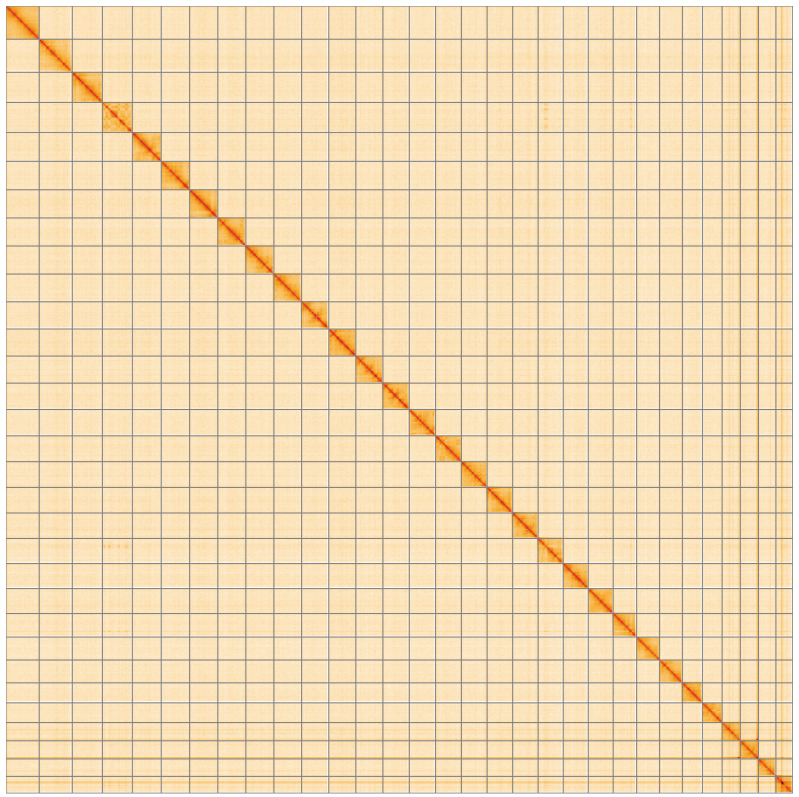
Genome assembly of *Xanthia icteritia*, ilXanIcte2.2: Hi-C contact map of the ilXanIcte2.2 assembly, visualised using HiGlass. Chromosomes are shown in order of size from left to right and top to bottom. An interactive version of this figure may be viewed at https://genome-note-higlass.tol.sanger.ac.uk/l/?d=RNncYqMFT8iWB2CPH557Ig.

The final assembly has a total length of 664.6 Mb in 36 sequence scaffolds with a scaffold N50 of 22.3 Mb ( Table 1). The snail plot in Figure 2 provides a summary of the assembly statistics, while the distribution of assembly scaffolds on GC proportion and coverage is shown in Figure 3. The cumulative assembly plot in Figure 4 shows curves for subsets of scaffolds assigned to different phyla. Most (99.96%) of the assembly sequence was assigned to 31 chromosomal-level scaffolds, representing 29 autosomes and the Z sex chromosome. Chromosome-scale scaffolds confirmed by the Hi-C data are named in order of size ( Figure 5; Table 2). While not fully phased, the assembly deposited is of one haplotype. Contigs corresponding to the second haplotype have also been deposited. The mitochondrial genome was also assembled and can be found as a contig within the multifasta file of the genome submission.

**Table 1.** Genome data for *Xanthia icteritia*, ilXanIcte2.2.

Project accession data		
Assembly identifier	ilXanIcte2.2	
Species	*Xanthia icteritia*	
Specimen	ilXanIcte2	
NCBI taxonomy ID	987434	
BioProject	PRJEB58663	
BioSample ID	SAMEA14448318	
Isolate information	ilXanIcte2, male: abdomen (DNA sequencing), head and thorax (Hi-C data)	
Assembly metrics *		*Benchmark*
Consensus quality (QV)	70.1	*≥ 50*
*k*-mer completeness	100.0%	*≥ 95%*
BUSCO **	C:98.9%[S:98.2%,D:0.7%],F:0.3%,M:0.8%,n:5,286	*C ≥ 95%*
Percentage of assembly mapped to chromosomes	99.96%	*≥ 95%*
Sex chromosomes	Z	*localised homologous pairs*
Organelles	Mitochondrial genome: 15.58 kb	*complete single alleles*
Raw data accessions		
PacificBiosciences Sequel IIe	ERR10753930	
Hi-C Illumina	ERR10742412	
Genome assembly		
Assembly accession	GCA_949128155.2	
*Accession of alternate haplotype*	GCA_949128055.1	
Span (Mb)	664.6	
Number of contigs	93	
Contig N50 length (Mb)	14.9	
Number of scaffolds	36	
Scaffold N50 length (Mb)	22.3	
Longest scaffold (Mb)	28.05	
Genome annotation		
Number of protein-coding genes	18,792	
Number of gene transcripts	18,983	

* Assembly metric benchmarks are adapted from column VGP-2020 of “Table 1: Proposed standards and metrics for defining genome assembly quality” from Rhie *et al.* (2021). ** BUSCO scores based on the lepidoptera_odb10 BUSCO set using version v5.4.3. C = complete [S = single copy, D = duplicated], F = fragmented, M = missing, n = number of orthologues in comparison. A full set of BUSCO scores is available at https://blobtoolkit.genomehubs.org/view/Xanthia_icteritia/dataset/GCA_949128155.2/busco.

**Table 2.** Chromosomal pseudomolecules in the genome assembly of *Xanthia icteritia*, ilXanIcte2.

INSDC accession	Chromosome	Length (Mb)	GC%
OX421930.2	1	27.92	38.0
OX421932.2	2	25.4	38.0
OX421933.2	3	25.3	38.0
OX421934.2	4	24.4	38.0
OX421935.2	5	24.01	37.5
OX421936.2	6	23.8	38.0
OX421937.2	7	23.69	37.5
OX421938.2	8	23.69	37.5
OX421939.2	9	23.38	38.0
OX421940.2	10	22.94	37.5
OX421941.2	11	22.87	38.0
OX421942.2	12	22.86	37.5
OX421943.2	13	22.25	37.5
OX421944.2	14	22.2	37.5
OX421945.2	15	21.81	38.0
OX421946.2	16	21.74	37.5
OX421947.2	17	21.59	37.5
OX421948.2	18	21.48	38.0
OX421949.2	19	21.2	38.0
OX421950.2	20	21.12	37.5
OX421951.2	21	21.08	38.0
OX421952.2	22	19.8	37.5
OX421953.2	23	19.4	38.0
OX421954.2	24	19.27	38.0
OX421955.2	25	16.86	38.0
OX421956.2	26	16.67	38.0
OX421957.2	27	15.4	39.0
OX421958.2	28	15.29	39.0
OX421959.2	29	14.73	38.5
OY855862.1	30	14.19	39.5
OX421931.1	Z	28.05	37.5
OX421960.1	MT	0.02	20.5

The estimated Quality Value (QV) of the final assembly is 70.1 with *k*-mer completeness of 100.0%, and the assembly has a BUSCO v completeness of 98.9% (single = 98.2%, duplicated = 0.7%), using the lepidoptera_odb10 reference set ( *n* = 5,286).

Metadata for specimens, BOLD barcode results, spectra estimates, sequencing runs, contaminants and pre-curation assembly statistics are given at https://links.tol.sanger.ac.uk/species/987434.

## Genome annotation report

The *Xanthia icteritia* genome assembly (GCA_949128155.1) was annotated at the European Bioinformatics Institute (EBI) on Ensembl Rapid Release. The resulting annotation includes 18,983 transcribed mRNAs from 18,792 protein-coding genes ( Table 1; https://rapid.ensembl.org/Xanthia_icteritia_GCA_949128155.1/Info/Index).

## Methods

### Sample acquisition and nucleic acid extraction

A male *Xanthia icteritia* (specimen ID NHMUK014451587, individual ilXanIcte2) was collected from Beinn Eighe National Nature Reserve, Scotland, UK (latitude 57.63, longitude –5.35) on 2021-09-09 using a light trap. The specimen was collected and identified by David Lees (Natural History Museum) and preserved at –80°C.

The workflow for high molecular weight (HMW) DNA extraction at the Wellcome Sanger Institute (WSI) Tree of Life Core Laboratory includes a sequence of core procedures: sample preparation; sample homogenisation, DNA extraction, fragmentation, and clean-up. In sample preparation, the ilXanIcte2 sample was weighed and dissected on dry ice ( Jay *et al.*, 2023). Abdomen tissue was homogenised using a PowerMasher II tissue disruptor ( Denton *et al.*, 2023a). HMW DNA was extracted using the Automated MagAttract v1 protocol ( Sheerin *et al.*, 2023). DNA was sheared into an average fragment size of 12–20 kb in a Megaruptor 3 system with speed setting 30 ( Todorovic *et al.*, 2023). Sheared DNA was purified by solid-phase reversible immobilisation ( Strickland *et al.*, 2023): in brief, the method employs a 1.8X ratio of AMPure PB beads to sample to eliminate shorter fragments and concentrate the DNA. The concentration of the sheared and purified DNA was assessed using a Nanodrop spectrophotometer and Qubit Fluorometer and Qubit dsDNA High Sensitivity Assay kit. Fragment size distribution was evaluated by running the sample on the FemtoPulse system.

Protocols developed by the WSI Tree of Life laboratory are publicly available on protocols.io ( Denton *et al.*, 2023b).

### Sequencing

Pacific Biosciences HiFi circular consensus DNA sequencing libraries were constructed according to the manufacturers’ instructions. DNA sequencing was performed by the Scientific Operations core at the WSI on a Pacific Biosciences Sequel IIe instrument. Hi-C data were also generated from head tissue of ilXanIcte2 using the Arima2 kit and sequenced on the Illumina NovaSeq 6000 instrument.

### Genome assembly and curation

Assembly was carried out with Hifiasm ( Cheng *et al.*, 2021) and haplotypic duplication was identified and removed with purge_dups ( Guan *et al.*, 2020). The assembly was then scaffolded with Hi-C data ( Rao *et al.*, 2014) using YaHS ( Zhou *et al.*, 2023). The assembly was checked for contamination and corrected using the gEVAL system ( Chow *et al.*, 2016) as described previously ( Howe *et al.*, 2021). Manual curation was performed using gEVAL, HiGlass ( Kerpedjiev *et al.*, 2018) and PretextView ( Harry, 2022). The mitochondrial genome was assembled using MitoHiFi ( Uliano-Silva *et al.*, 2023), which runs MitoFinder ( Allio *et al.*, 2020) or MITOS ( Bernt *et al.*, 2013) and uses these annotations to select the final mitochondrial contig and to ensure the general quality of the sequence.

### Final assembly evaluation

The final assembly was post-processed and evaluated with the three Nextflow ( Di Tommaso *et al.*, 2017) DSL2 pipelines “sanger-tol/readmapping” ( Surana *et al.*, 2023a), “sanger-tol/genomenote” ( Surana *et al.*, 2023b), and “sanger-tol/blobtoolkit” ( Muffato *et al.*, 2024). The pipeline sanger-tol/readmapping aligns the Hi-C reads with bwa-mem2 ( Vasimuddin *et al.*, 2019) and combines the alignment files with SAMtools ( Danecek *et al.*, 2021). The sanger-tol/genomenote pipeline transforms the Hi-C alignments into a contact map with BEDTools ( Quinlan & Hall, 2010) and the Cooler tool suite ( Abdennur & Mirny, 2020), which is then visualised with HiGlass ( Kerpedjiev *et al.*, 2018). It also provides statistics about the assembly with the NCBI datasets ( Sayers *et al.*, 2024) report, computes *k*-mer completeness and QV consensus quality values with FastK and MerquryFK, and a completeness assessment with BUSCO ( Manni *et al.*, 2021).

The sanger-tol/blobtoolkit pipeline is a Nextflow port of the previous Snakemake Blobtoolkit pipeline ( Challis *et al.*, 2020). It aligns the PacBio reads with SAMtools and minimap2 ( Li, 2018) and generates coverage tracks for regions of fixed size. In parallel, it queries the GoaT database ( Challis *et al.*, 2023) to identify all matching BUSCO lineages to run BUSCO ( Manni *et al.*, 2021). For the three domain-level BUSCO lineage, the pipeline aligns the BUSCO genes to the Uniprot Reference Proteomes database ( Bateman *et al.*, 2023) with DIAMOND ( Buchfink *et al.*, 2021) blastp. The genome is also split into chunks according to the density of the BUSCO genes from the closest taxonomically lineage, and each chunk is aligned to the Uniprot Reference Proteomes database with DIAMOND blastx. Genome sequences that have no hit are then chunked with seqtk and aligned to the NT database with blastn ( Altschul *et al.*, 1990). All those outputs are combined with the blobtools suite into a blobdir for visualisation.

All three pipelines were developed using the nf-core tooling ( Ewels *et al.*, 2020), use MultiQC ( Ewels *et al.*, 2016), and make extensive use of the Conda package manager, the Bioconda initiative ( Grüning *et al.*, 2018), the Biocontainers infrastructure ( da Veiga Leprevost *et al.*, 2017), and the Docker ( Merkel, 2014) and Singularity ( Kurtzer *et al.*, 2017) containerisation solutions.

Table 3 contains a list of relevant software tool versions and sources.

**Table 3.** Software tools: versions and sources.

Software tool	Version	Source
BEDTools	2.30.0	https://github.com/arq5x/bedtools2
Blast	2.14.0	ftp://ftp.ncbi.nlm.nih.gov/blast/executables/blast+/
BlobToolKit	4.3.7	https://github.com/blobtoolkit/blobtoolkit
BUSCO	5.4.3 and 5.5.0	https://gitlab.com/ezlab/busco
bwa-mem2	2.2.1	https://github.com/bwa-mem2/bwa-mem2
Cooler	0.8.11	https://github.com/open2c/cooler
DIAMOND	2.1.8	https://github.com/bbuchfink/diamond
fasta_windows	0.2.4	https://github.com/tolkit/fasta_windows
FastK	427104ea91c78c3b8b8b49f1a7d6bbeaa869ba1c	https://github.com/thegenemyers/FASTK
GoaT CLI	0.2.5	https://github.com/genomehubs/goat-cli
Hifiasm	0.16.1-r375	https://github.com/chhylp123/hifiasm
HiGlass	44086069ee7d4d3f6f3f0012569789ec138f42b84aa44357826c0b6753eb28de	https://github.com/higlass/higlass
MerquryFK	d00d98157618f4e8d1a9190026b19b471055b22e	https://github.com/thegenemyers/MERQURY.FK
MitoHiFi	2	https://github.com/marcelauliano/MitoHiFi
MultiQC	1.14, 1.17, and 1.18	https://github.com/MultiQC/MultiQC
NCBI Datasets	15.12.0	https://github.com/ncbi/datasets
Nextflow	23.04.0-5857	https://github.com/nextflow-io/nextflow
PretextView	0.2	https://github.com/wtsi-hpag/PretextView
purge_dups	1.2.3	https://github.com/dfguan/purge_dups
samtools	1.16.1, 1.17, and 1.18	https://github.com/samtools/samtools
sanger-tol/genomenote	1.1.1	https://github.com/sanger-tol/genomenote
sanger-tol/readmapping	1.2.1	https://github.com/sanger-tol/readmapping
Seqtk	1.3	https://github.com/lh3/seqtk
Singularity	3.9.0	https://github.com/sylabs/singularity
YaHS	yahs-1.1.91eebc2	https://github.com/c-zhou/yahs

### Genome annotation

The BRAKER2 pipeline ( Brůna *et al.*, 2021) was used in the default protein mode to generate annotation for the *Xanthia icteritia* assembly (GCA_949128155.1) in Ensembl Rapid Release at the EBI.

### Wellcome Sanger Institute – Legal and Governance

The materials that have contributed to this genome note have been supplied by a Darwin Tree of Life Partner. The submission of materials by a Darwin Tree of Life Partner is subject to the **‘Darwin Tree of Life Project Sampling Code of Practice’**, which can be found in full on the Darwin Tree of Life website here. By agreeing with and signing up to the Sampling Code of Practice, the Darwin Tree of Life Partner agrees they will meet the legal and ethical requirements and standards set out within this document in respect of all samples acquired for, and supplied to, the Darwin Tree of Life Project.

Further, the Wellcome Sanger Institute employs a process whereby due diligence is carried out proportionate to the nature of the materials themselves, and the circumstances under which they have been/are to be collected and provided for use. The purpose of this is to address and mitigate any potential legal and/or ethical implications of receipt and use of the materials as part of the research project, and to ensure that in doing so we align with best practice wherever possible. The overarching areas of consideration are:

•   Ethical review of provenance and sourcing of the material

•   Legality of collection, transfer and use (national and international)

Each transfer of samples is further undertaken according to a Research Collaboration Agreement or Material Transfer Agreement entered into by the Darwin Tree of Life Partner, Genome Research Limited (operating as the Wellcome Sanger Institute), and in some circumstances other Darwin Tree of Life collaborators.

## Data Availability

European Nucleotide Archive: *Xanthia icteritia*. Accession number PRJEB58663; https://identifiers.org/ena.embl/PRJEB58663 ( Wellcome Sanger Institute, 2023). The genome sequence is released openly for reuse. The *Xanthia icteritia* genome sequencing initiative is part of the Darwin Tree of Life (DToL) project. All raw sequence data and the assembly have been deposited in INSDC databases. Raw data and assembly accession identifiers are reported in Table 1. Members of the Natural History Museum Genome Acquisition Lab are listed here: https://doi.org/10.5281/zenodo.7139035. Members of the Darwin Tree of Life Barcoding collective are listed here: https://doi.org/10.5281/zenodo.4893703. Members of the Wellcome Sanger Institute Tree of Life Management, Samples and Laboratory team are listed here: https://doi.org/10.5281/zenodo.10066175. Members of Wellcome Sanger Institute Scientific Operations: Sequencing Operations are listed here: https://doi.org/10.5281/zenodo.10043364. Members of the Wellcome Sanger Institute Tree of Life Core Informatics team are listed here: https://doi.org/10.5281/zenodo.10066637. Members of the Tree of Life Core Informatics collective are listed here: https://doi.org/10.5281/zenodo.5013541. Members of the Darwin Tree of Life Consortium are listed here: https://doi.org/10.5281/zenodo.4783558.
