# Supplementary material for: The genome sequence of the Sallow moth, Xanthia icteritia (Hufnagel, 1766)
Source: Wellcome Open Res. 2024 May 15;9:269. [Version 1] doi: 10.12688/wellcomeopenres.21623.1 (PMC12598659; doi:10.12688/wellcomeopenres.21623.1)
Supplement: Peer review history

# Reviewer report: Reviewer response for version 1

**DOI:** 10.21956/wellcomeopenres.23895.r134378

**Published:** 2025-11-08

**Authors:** Sundaram Janarthanan

**Affiliations:** 1 University of Madras, Chennai, Tamil Nadu, India

As an economically important insect species, the genome sequence of Xanthia icteritia will be a valuable resource for future reference. Furthermore, the genome assembly was annotated with 18,792 protein-coding genes, which is particularly useful for comparative analysis for any future research related to functional studies. Hence, I recommend its indexing in the Tree of Life..

Are sufficient details of methods and materials provided to allow replication by others?

Yes

Is the rationale for creating the dataset(s) clearly described?

Yes

Are the datasets clearly presented in a useable and accessible format?

Yes

Are the protocols appropriate and is the work technically sound?

Yes

Reviewer Expertise:

Insect Physiology, Biochemistry and Molecular Biology.

I confirm that I have read this submission and believe that I have an appropriate level of expertise to confirm that it is of an acceptable scientific standard.

# Reviewer report: Reviewer response for version 1

**DOI:** 10.21956/wellcomeopenres.23895.r98001

**Published:** 2024-10-02

**Authors:** Merly Escalona

**Affiliations:** 1 University of California Santa Cruz, Santa Cruz, California, USA

The authors present the Sallow moth's high-quality chromosome-level genome assembly. The genome was assembled using PacBio HiFi long reads and Hi-C data, followed by manual curation. The methods used for data generation, processing, assembly, and quality checks are well-documented and described adequately. Assembly metrics satisfy the benchmarks for the VGP-2020 baseline (Rhie et al. 2021). The metadata provided are comprehensive, and the genome resource has a clear and direct application. 

**Minor comments:**

- Background. Paragraph 1. First sentence: 
  - The Sallow, *Xanthia icteritia *(Hufnagel, 1766), sometimes placed in the genus or subgenus *Cirrhia* (Hübner, 1821), of which it is the type species, is a moth of the family Noctuidae.
  - Could be rewritten as:
    - The Sallow, *Xanthia icteritia* (Hufnagel, 1766), is a moth of the family Noctuidae. However, it is sometimes placed in the genus or subgenus *Cirrhia* (Hübner, 1821), of which it is the type species.
- Background. Paragraph 3. Last sentence: 
  - …characterized as ‘severe’ the 81% decline in abundance abundance in..
  - Change to:
    -  …characterized as ‘severe’ the 81% decline in abundance in.
- Genome sequence report: Paragraph 2. Sentence:
  - Most (99.96%) of the assembly sequence was assigned to 31 chromosomal-level scaffolds, representing 29 autosomes and the Z sex chromosome.
  - Given that this is a male sample and the karyotype is ZZ, are both Z copies kept on the final assembly? Or did you mean 30 chromosomal-level scaffolds?
- What metrics are used to evaluate the completeness and correctness of the mitochondrial genome? Are the annotation metrics only for the nuclear genome? Both?

Are sufficient details of methods and materials provided to allow replication by others?

Yes

Is the rationale for creating the dataset(s) clearly described?

Yes

Are the datasets clearly presented in a useable and accessible format?

Yes

Are the protocols appropriate and is the work technically sound?

Yes

Reviewer Expertise:

genome assembly, bioinformatics

I confirm that I have read this submission and believe that I have an appropriate level of expertise to confirm that it is of an acceptable scientific standard.
